# PT Achievement in Public Hospitals and Its Effect on Outcomes

**DOI:** 10.3390/geriatrics4040058

**Published:** 2019-10-18

**Authors:** Jessica S. Morton, Alex Tang, Michael J. Moses, Dustin Hamilton, Neville Crick, Ran Schwarzkopf

**Affiliations:** NYU Langone Orthopedic Hospital, New York, NY 10003, USA; jessica.morton@nyulangone.org (J.S.M.); Alex.tang@nyulangone.org (A.T.); Michael.moses@nyulangone.org (M.J.M.); Hunterhamiltonmed@gmail.com (D.H.); Neville.Crick@nyulangone.org (N.C.)

**Keywords:** physical therapy, discharge, total knee arthroplasty, length of stay

## Abstract

The demand for TKA continues to rise within the United States, while increasing quality measures and cost containment became the basis of reimbursement for hospital systems. Length of stay is a major driver in the cost of TKA. Early mobilization with physical therapy has been shown to increase range of motion and decrease complications, but with mixed results in regards to length of stay. We postulate that initiating physical therapy on post-operative day zero will decrease length of stay in an urban public hospital. Retrospective chart review was performed at a large, urban, public academic medical center to identify patients who have had a primary TKA over the course of a 3-year period. Groups who underwent post-operative day zero therapy were compared with those who initiated physical therapy on post-operative day one. Length of stay was the primary outcome. Patient demographic characteristics and discharge disposition were also collected. There were 98 patients in the post-operative day-one physical therapy cohort and 58 in the post-operative day zero physical therapy group. Hospital length of stay was significantly decreased in the post-operative day zero physical therapy group. (*p* < 0.01) There was no difference in discharge disposition between the two groups.

## 1. Introduction

The demand for total knee arthroplasty (TKA) in the United States is increasing annually and projected to rise to 3.48 million annually by 2030 [1]. The increase in volume of procedures is secondary to increased diagnosis of degenerative joint disease, with an increased demand for function and mobility in a population with increased life expectancy [1]. While TKA remains a cost-effective procedure [2], the rising numbers of TKA generates significant healthcare expenditure. In order to curb costs while maintaining patient outcomes, focus on quality metrics has become a priority of both the insurance companies and hospital systems. The Center for Medicare and Medicaid Services (CMS) has utilized the following quality metrics to guide this new value-based system: readmissions within 90 days of the operation, complications, and hospital length of stay (LOS).

Hospital length of stay is a major driver of cost to the healthcare system in patients who undergo total knee arthroplasty [3,4]. The average daily hospital inpatient cost has doubled to over two thousand dollars a day in 2017 [5]. Length of stay has decreased significantly over the past decade with the use of clinical pathways; however, the average length of stay after TKA in the Medicare population is reported at 3.7 days [4,6,7]. Despite this length of stay CMS recently removed TKA from the Inpatient Only (IPO) list. Removal of TKA from the inpatient only list creates additional pressure on hospital systems to reduce length of stay. Not-for-profit safety net hospitals face additional cost pressures and single digit operating margins [8]. 

Numerous studies have established patient and surgical factors that affect LOS including age, gender, medical comorbidities, anesthetic type, blood transfusion, and surgical day of the week [9,10,11,12,13,14]. Physical therapy (PT) is a pillar of postoperative care following TKA. Physical therapy has been shown to increase range of motion (ROM), decrease swelling, improve strength, and improve level of function after TKA. However the literature remains mixed in regards to whether physical therapy impacts length of stay for TKA patients [15,16,17]. 

The purpose of this study is to investigate the effect of same day physical therapy on length of stay following primary TKA in a single, large, urban public hospital in the United States. 

## 2. Materials and Methods

### 2.1. Design and Study Population

A retrospective chart review was performed at a large, urban, public academic medical center to identify patients who have had a primary TKA over the course of a 3-year period (2014–2017). The institution performed elective total joint arthroplasty one day per week throughout the course of the study period. We collected length of stay, whether the patient received post-operative day zero (POD-0) PT or post-operative day one (POD-1) PT, baseline patient demographics (i.e., age, gender, body mass index (BMI), American Society of Anesthesiologists (ASA) score, and race), and discharge disposition. Inclusion criteria included patients who were greater than or equal to 18-years-old and were undergoing elective primary TKAs. Exclusion criteria included any patients less than 18-years-old at the time of the surgery, revision surgery, infection and patients undergoing non-elective TKAs (e.g., acute trauma to the joint or revision arthroplasty). 

### 2.2. Therapy Protocol

Patients received the same therapy protocol regardless of day of initiation of therapy. Physical therapy was conducted once on POD-0 for those in the POD-0 group and one to two times daily thereafter for both groups under the direct supervision of a physical therapist. A detailed description of the graduated therapy protocol can be found in Table 1. Patients were deemed safe for discharge if they attained standardized goals: (1) Independent or modified independent transfers and bed mobility, (2) independent ambulation 50 feet with walker or cane if a household ambulator, 100 feet if a community ambulator, and (3) knee flexion of 70–90°.

### 2.3. Statistical Analyses

Data was collected and de-identified using Microsoft Excel software. All statistical analyses and calculations were conducted using SPSS v23 software. A simple Poisson regression and Fisher’s exact test were used for simple bivariate comparisons between length of stay or baseline patient demographics and same-day PT. A multivariable Poisson regression was used to compare length of stay, baseline patient demographics, and same-day PT. A *p*-value < 0.05 was used to determine statistical significance of a finding.

## 3. Results

From January 2014 to May 2017 we identified 156 patients who underwent primary TKA at Bellevue Hospital and met our inclusion criteria. Ninety-eight patients received physical therapy on postoperative day one (POD-1), fifty-eight patients received physical therapy on POD-0. There were no significant differences in age, sex, BMI, ASA class, or race between the two groups. Detailed demographic data can be found in Table 2. 

Length of stay was significantly decreased for the POD-0 physical therapy group compared with those receiving physical therapy on POD-1.Average length of stay for patients receiving physical therapy on POD-1 was 3.97 days (±2.66), the average length of stay for patients receiving physical therapy on POD-0 was 2.05 (±1.09), Female gender was associated with a significantly increased length of stay. This was found to be significant with multivariable regression analysis Multiregression analysis can be found in Table 3. No other demographic factors studied demonstrated a change in patient length of stay.

There were no differences in the discharge disposition of patients as determined by POD-0 physical therapy p = 0.286. There was a trend towards increased discharge to home, but did not reach clinical significance. Table 4 details discharge disposition. 

## 4. Discussion

Safe ambulation after TKA is a major determinant of discharge disposition and hospital length of stay. Post-operative day zero physical therapy after TKA has been proven to be safe [17,18]. Numerous studies have shown that physical therapy is beneficial for range of motion, short term function, and prevention of complications [19,20].

However, there are conflicting reports of the effect of early postoperative rehab for TKA. Karim and colleagues found that POD-0 PT did not affect LOS in TKA but did in THA [17]. While our study has shown our intensive physical therapy program beginning POD-0 decreases hospital length of stay regardless of patient comorbidities or age. This is similar to Labraca et al., who found significantly shorter hospital stay, fewer rehabilitation sessions until discharge, less pain, greater joint range of motion in flexion and extension, improved quad and hamstring strength and higher gait and balance scores [21]. Isaac and colleagues used a multimodal model in their post-operative patients, utilizing analgesia and team anticipation of discharge plans as well as accelerated PT, which resulted in a reduced mean length of hospital stay after surgery to 3.6 (S.D. 1.0) days, from a previous departmental average of 10.5 days [22]. 

The present study supports the findings of Chen and Den Hertog, who found decreased length of stay with initiation of physical therapy on POD-0 [18,23]. However, it conflicts with the findings of Bohl et al. who conducted a randomized controlled trial of 394 patients undergoing TKA randomized to physical therapy on POD-1 vs POD-0 and found no significant differences in length of stay, patent-reported readiness for discharge, or pain. Bohl raises the contention that previous studies have selection bias leading to same day physical therapy as the patients not having POD-0 therapy were due to case timing, staff availability, or postoperative factors such as dizziness, nausea, and pain which is a potential confounder of our study [24]. However, this does represent real life conditions and may assist in physicians in patient counseling and expectations, if they are unable to perform PT on POD-0 it may provide an early indicator of an increased length of stay. 

This study did not find a significant difference in discharge destination for patients who underwent initiation of therapy on POD-0-. There was a trend towards increased discharge home however the difference was not statistically significant. Sarapong et al and others found that patient were significantly less likely to be discharged to a rehabilitation facility when initiating physical therapy on POD-0 [25,26,27,28] The lack of difference in discharge destination may be secondary to patient social factors in our public hospital in which many patients in both groups live in apartments with multiple flights of stairs and are unable to safely be discharged home in the immediate postoperative period. 

An intensive physical therapy protocol with initiation of physical therapy POD-0-, and one to two daily sessions was examined by Moses et al [29]. While two physical therapy sessions a day is an increased demand on staff it resulted in decreased length of stay, ultimately decreasing cost. Length of stay is a major driver of cost following TKA. The ability to decrease cost in a public hospital without sacrificing patient satisfaction or readiness for discharge is increasingly important in public hospitals with tight operating margins [8]. The additive cost of reducing inpatient admissions by an entire day is beneficial to the hospital and healthcare system. 

This study has several limitations including those inherent to its retrospective design, potentially exposing the study to selection bias as patients where not randomized to receive PT on POD -1 or POD-0 but rather naturally occurred secondary to case timing, patient readiness, and staff availability. Furthermore, retrospective design exposes the study to potential confounding variables including institutional changes over the course of the study period. Another limitation is the lack of validated patient outcome score both pre and postoperatively. A prospective design in which these variables as well as longer term patient outcomes and complications may benefit the literature. Despite these limitations, the study indicates that initiating PT on postoperative day zero can reduce length of stay by a significant factor regardless of patient baseline demographics.

## 5. Conclusions

Initiating Physical Therapy on POD-0 decreases hospital Length of Stay by almost a full day regardless of age, race, sex, and pre-existing comorbidities. Initiating POD-0 physical therapy significantly decreased length of stay and trended towards increased discharge to home after TKA. 

## Figures and Tables

**Table 1:** Physical Therapy Protocol.

Illustrated Exercise Program Provided
Gastrocnemius stretching, ankle Pumps Quadriceps, Hamstring isometric stretching
Active assist knee flexion and extension Supervised out of bed to chair transfers Supervised ambulation for 50 feet with assistive device
Supervised stair negotiation 4-6 steps, step to step pattern Independent out of bed to chair transfers Modified independent ambulation for 100 feet with assistive device

**Table 2:** Bivariate comparisons of length of stay and patient characteristics by same-day PT, represented as counts (%) or mean (±SD) *.

Variables	No Same-Day PT (n = 98)	Same-Day PT (n = 58)	*p*-Value **
LOS	3.97 (±2.66)	2.05 (±1.09)	< 0.01
Age Group			0.80
0–59	25 (25.5)	15 (25.9)	
60–64	21 (21.4)	16 (27.6)	
65–70	21 (21.4)	12 (20.7)	
71+	31 (31.6)	15 (25.9)	
Sex			0.70
Male	22 (22.4)	15 (25.9)	
Female	76 (77.6)	43 (74.1)	
BMI			0.76
Normal, <25	11 (11.2)	8 (13.8)	
Overweight 25–29.9	24 (24.5)	16 (27.6)	
Obese 30+	63 (64.3)	34 (58.6)	
ASA			0.23
1 &2	86 (88.7)	46 (80.7)	
3	11 (11.3)	11 (19.3)	
Race			0.33
Hispanic	47 (49.0	29 (60.4)	
White	9 (9.4)	3 (6.2)	
Black	28 (29.2)	8 (16.7)	
Asian	12 (12.5)	8 (16.7)	

* Not all counts sum to sample total due to missing data for some variables. ** *p*-Values are derived from simple Poisson regression for LOS and Fisher’s exact test for categorical variables.

**Table 3:** Multivariable Poisson regression for length of stay predicted by same-day physical therapy and patient characteristics.

Variables	IRR (95% CI)	*p*-Value
Same-Day PT	0.54 (0.43, 0.68)	<0.01
Age Group		
0–59 (ref)	---	---
60–64	1.09 (0.83, 1.45)	0.52
65–70	1.04 (0.78, 1.39)	0.79
71+	1.25 (0.97, 1.62)	0.09
Sex		
Male (ref)	---	---
Female	1.38 (1.08, 1.77)	0.01
BMI		
Normal, <25 (ref)	---	---
Overweight 25–29.9	0.88 (0.63, 1.22)	0.44
Obese 30+	1.05 (0.78, 1.43)	0.74
ASA		
1 &2 (ref)	---	---
3	0.99 (0.71, 1.37)	0.95
Race		
Hispanic (ref)	---	---
White	1.29 (0.95, 1.77)	0.11
Black	0.98 (0.78, 1.23)	0.85
Asian	1.20 (0.90, 1.60)	0.21

**Table 4:** Bivariate comparisons of disposition status by same-day PT, represented as counts (%).

Variables	No Same-Day PT (n = 96)	Same-Day PT (n = 57)	*p*-Value
**Disposition Status**			0.28612034
Subacute Rehab (SAR)	18 (19)	6 (11)	
Skilled Nursing Facility (SNF)	1 (1)	0 (0)	
Home	77 (80)	51 (89)	
